# Pipeline Embolization Device for the Treatment of Ruptured Intracerebral Aneurysms: A Multicenter Retrospective Study

**DOI:** 10.3389/fneur.2021.675917

**Published:** 2021-09-16

**Authors:** Weiying Zhong, Hong Kuang, Ping Zhang, Xinjian Yang, Bin Luo, Aisha Maimaitili, Yuanli Zhao, Donglei Song, Sheng Guan, Hongqi Zhang, Yang Wang, Donghai Wang, Wandong Su, Yunyan Wang

**Affiliations:** ^1^Department of Neurosurgery, Institute of Brain and Brain-Inspired Science, Qilu Hospital of Shandong University, Shandong University, Jinan, China; ^2^Shandong Key Laboratory of Brain Function Remodeling, Shandong University, Jinan, China; ^3^Department of Neurosurgery, The Second Affiliated Hospital of Guangxi Medical University, Nanning, China; ^4^Beijing Neurosurgical Institute, Beijing Tiantan Hospital, Capital Medical University, Beijing, China; ^5^First Affiliated Hospital of Xinjiang Medical University, Urumqi, China; ^6^Peking University International Hospital, Beijing, China; ^7^Shanghai Donglei Brain Hospital, Shanghai, China; ^8^First Affiliated Hospital of Zhengzhou University, Zhengzhou, China; ^9^Xuanwu Hospital, Capital Medical University, Beijing, China; ^10^First Affiliated Hospital of Nanchang University, Nanchang, China

**Keywords:** intracerebral aneurysm, pipeline embolization device, flow diversion, subarachnoid hemorrhage, complication

## Abstract

**Background and Purpose:** The utilization of flow diversion for ruptured intracerebral aneurysms (IAs) is still limited. We aimed to demonstrate our multicenter experience using the pipeline embolization device (PED) for ruptured IAs that were difficult to treat by clipping and coiling.

**Methods:** Thirty-eight patients with ruptured IAs who underwent PED treatment from 2015 to 2020 were retrospectively reviewed. Factors associated with procedure-related stroke (ischemic and hemorrhagic) and clinical and angiography outcomes were analyzed.

**Results:** There were 14 (36.8%) saccular IAs, 12 (31.6%) blister-like IAs, and 12 (31.6%) dissecting IAs. Perforator involvement was noted in 10 (26.3%) IAs. Early PED placement ( ≤ 15 days) and adjunctive coiling treatment were performed in 27 (71.1%) and 22 (57.9%) cases, respectively. The overall rate of stroke-related complications was 31.6% (12/38) (including rates of 10.5% for procedure-related hemorrhagic complications and 15.8% for procedure-related infarction). The mortality rate was 13.2% (5/38), and 84.2% of patients (32/38) had favorable outcomes. Thirty-two (84.2%) patients underwent follow-up angiographic evaluations; of these, 84.4% (27 patients) had complete occlusion and 15.6% had incomplete obliteration. Multivariate analysis revealed that early PED placement was not associated with a high risk of procedure-related stroke or an unfavorable outcome. Adjunctive coiling exhibited an association with procedure-related stroke (*p* = 0.073). Procedure-related hemorrhagic complications were significantly associated with an unfavorable outcome (*p* = 0.003). Immediate contrast stasis in the venous phase was associated with complete occlusion during follow-up (*p* = 0.050).

**Conclusion:** The PED is a feasible and effective treatment to prevent rebleeding and achieve aneurysm occlusion, but it is associated with a substantial risk of periprocedural hemorrhage and ischemic complications in acute ruptured IAs. Therefore, the PED should be used selectively for acutely ruptured IAs. Additionally, adjunctive coiling might increase procedure-related stroke; however, it may reduce aneurysm rebleeding in acutely ruptured IAs. Patients with immediate contrast stasis in the venous phase were more likely to achieve total occlusion. A prospective study with a larger sample size should be performed to verify our results.

## Introduction

Flow diverters such as the pipeline embolization device (PED) have been widely used to treat non-ruptured, complex, intracerebral aneurysms (IAs). These devices have been shown to be safe and efficient, and their indications have been gradually extended (1, 2). However, PEDs have not been widely used to treat ruptured IAs. Some ruptured IAs are challenging for conventional endovascular and neurosurgical treatment; therefore, previous studies have reported their limited experiences regarding the utilization of flow diversion for challenging ruptured IAs, including giant, dissecting, or blister aneurysms (3–5). Those previous studies indicated that flow diverter deployment was feasible but more challenging for ruptured IAs, and patients may experience more complications than patients with unruptured IAs (6). However, these previous studies often included few cases using different flow diverters and strategies, such as flow diversion with or without additional coiling or coiling first followed by staged flow diversion, and the results were heterogeneous. Therefore, use of flow diversion in ruptured IAs requires further study. Compared with other countries, the application of flow diverters in aneurysm treatment is still rare and has not been widely used in China. Large studies examining treatment of ruptured aneurysms with flow diverters in the Chinese population are still lacking. Regional and ethnic differences in mortality following subarachnoid hemorrhage (SAH) have been demonstrated (7, 8); therefore, the results of flow diversion for ruptured aneurysms in Chinese patients may be inconsistent with the results from other countries. The PED is the first and most widely used flow diverter in China. Herein, we present our initial multicenter experience using the PED for treatment of ruptured IAs that are challenging to treat by clipping and coiling and focus on the procedure-related hemorrhagic and ischemic complications and clinical and angiographic outcomes.

## Materials and Methods

### Patients

The data supporting the findings of this study are available from the corresponding author upon reasonable request. From October 2015 to July 2020, 38 consecutive ruptured IAs, primarily treated with PEDs in eight Chinese centers, were included in this retrospective study. Aneurysms with arteriovenous malformation and those treated with coiling or stents before PED deployment (staged treatment) were excluded from this study. Medical records including the demographic data, medical comorbidities, initial neurological conditions, amount of SAH, morphological characteristics of the aneurysm, time to PED deployment, treatment modality (flow-diverting stents with or without adjunctive coiling), complications, and clinical and angiographic outcomes after treatment were obtained.

SAH was confirmed by computed tomography scans, and aneurysms were confirmed by digital subtraction angiography. The patient's initial neurological condition was evaluated using the Hunt and Hess (H&H) grading system and was classified as either mild (H&H grades 1–3) or serious (H&H grade 4 or 5). The SAH grade was classified according to the modified Fisher scale (mFS) as either a lesser hemorrhage (mFS 1 or 2) or a massive hemorrhage (mFS 3 or 4) (9). Aneurysms involving a perforating artery arising from the aneurysm neck, body, or dome were considered as having perforator involvement. Complications during hospitalization were divided into two subgroups: stroke-related (hemorrhage or infarction) and non-stroke-related complications. Procedural complications were considered as those that occurred intraoperatively or within 12 h after PED deployment, including stroke- and non-stroke-related complications. Clinical follow-up was performed via clinical examination or telephone interview, with a median follow-up time of 12 months (range, 3–65 months) in surviving patients. Clinical outcomes at the final follow-up were assigned according to the modified Rankin Scale (mRS), with a score of 0–2 considered as a favorable outcome and a score of 3–6 considered as an unfavorable outcome. Radiological follow-up by CT angiography, magnetic resonance angiography, or digital subtraction angiography was conducted at 3, 6, 12, and 24 months after the procedure. If patients had new clinical symptoms during follow-up, radiological follow-up was advanced. Aneurysm occlusion on the final follow-up angiogram was categorized as complete (100%) or incomplete (<100%).

### Procedure Details

**Figure 1:**
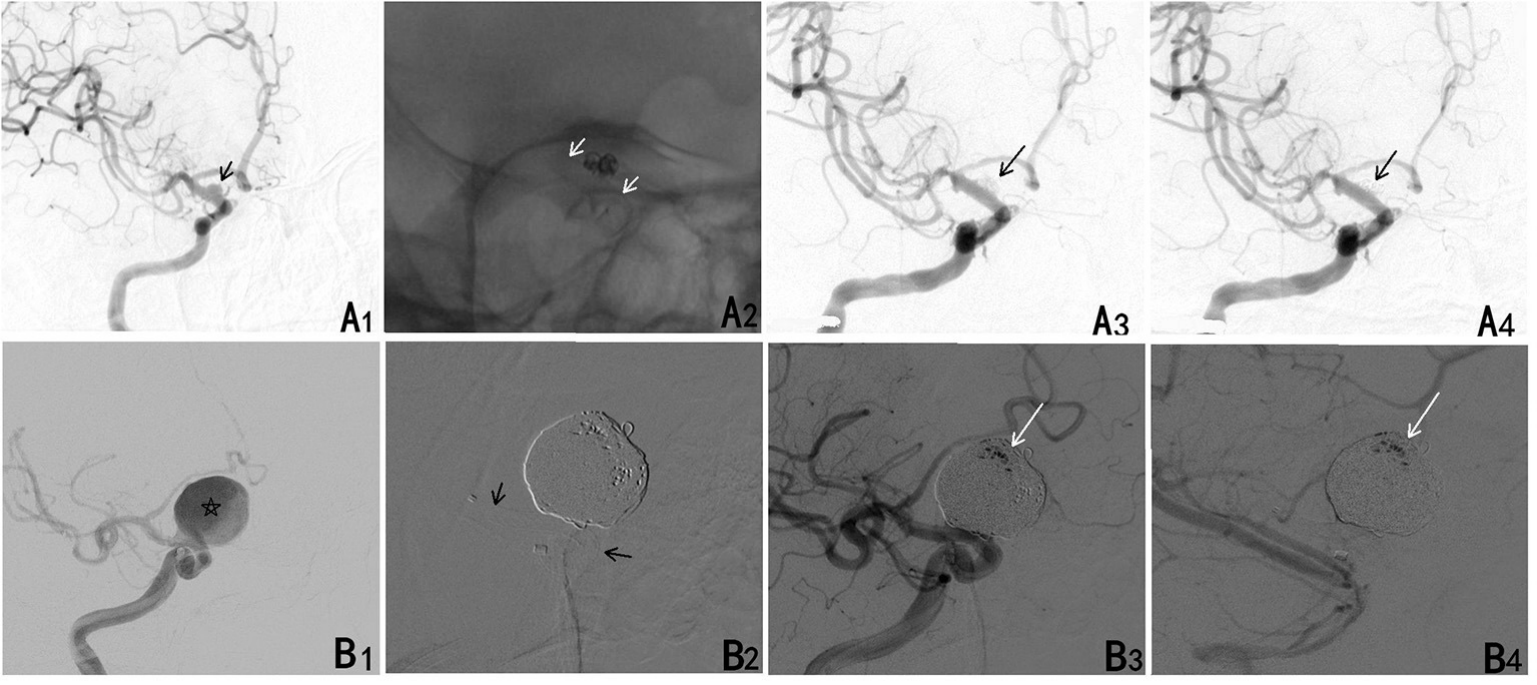
**(A)** A ruptured blood blister aneurysm treated with a PED. (A1) A ruptured blood blister aneurysm of the internal carotid artery (ICA) (black arrow). (A2) The aneurysm was treated with loosing packed coiling and a PED (white arrow). (A3) The aneurysm continued to fill completely in the arterial phase. (A4) The contrast cleared within the arterial phase. **(B)** A larger ruptured aneurysm treated with a PED. (B1) A ruptured saccular aneurysm of the ICA (star). (B2) The aneurysm was treated with densely packed coiling and a PED (black arrow). (B3) The aneurysm continued to fill incompletely in the arterial phase. (B4) Contrast stasis persisted through the venous phases.

In this study, PED use was evaluated by a multidisciplinary team including neurosurgeons and neuroradiologists at each center. Early PED treatment was considered as PED deployment within 15 days after SAH. The PED was utilized as the primary treatment modality to secure ruptured IAs with or without adjunctive coiling during the interventional procedure. The diameter and length of the PED were chosen according to the size of the aneurysm and parent vessel. A loading dose of aspirin (300 mg) and clopidogrel (300 mg) was administered 2–8 h prior to the procedure. An intravenous bolus of heparin (5,000 IU) was administered, and the activated clotting time was controlled between 250 and 300 s during the procedure. Heparin was discontinued at the end of the procedure. All endovascular procedures were performed *via* a femoral artery approach under general anesthesia. A standard 6-F or 8-F guide catheter was inserted into the individual target vessel, internal carotid artery, or vertebral artery. The parent vessel was then accessed through an intermediate catheter (Navien 058, Medtronic, Irvine, CA, USA). The PED was deployed through a Marksman microcatheter and covered the neck of the aneurysm. For aneurysms treated with additional coiling, a microcatheter was first placed into the aneurysm sac before PED deployment. The PED device was then partially deployed to cover the aneurysm neck and temporarily jail the microcatheter. The remainder of the stent was deployed after loose- or dense-packed coiling. The degree of angiographic occlusion was recorded using the O'Kelly–Marotta grading scale immediately after the procedure and again upon follow-up imaging (10). The degree of aneurysm filling was graded as follows: A, complete (>95%); B, incomplete (5–95%); C, neck remnant (<5%); or D, no filling (0%). Contrast stasis was graded as follows: 1, no stasis (clearance within the arterial phase); 2, moderate stasis (clearance prior to the venous phase); and 3, significant stasis (contrast persisted in the aneurysm during the venous phase and beyond; Figure 1). The O'Kelly–Marotta score was dichotomized into grades A–B vs. C–D or 1–2 vs. 3. The effectiveness of the antiplatelet regimen was evaluated by a thromboelastogram performed after the procedure for elective cases. Patients usually continually received dual antiplatelet therapy with clopidogrel (75 mg daily) for 3–6 months and aspirin (100 mg daily) ≥6 months after PED deployment. An insufficient response to either drug was treated by dose escalation or substitution with ticagrelor (90 mg twice daily).

### Statistical Analysis

SPSS 23.0 software (IBM Corp., Armonk, New York, USA) was used for the statistical analysis. Data are presented as the median (interquartile range) for continuous variables and frequency for categorical variables. During the statistical analysis, continuous variables such as age were transformed into categorical variables, and the cutoff point for continuous variables was their median. Fisher's exact test or the Pearson χ^2^ test was used to determine predictive factors. Factors with *p* < 0.1 in the univariate analysis were considered potential independent variables and subsequently included in the multivariate logistic regression analysis using a forward process. A *p*-value of < 0.05 was considered statistically significant, and *p*-values between 0.05 and 0.10 were considered to indicate a trend.

## Results

### Patients and Aneurysm Characteristics

The patients included 16 men and 22 women with a median age of 51 years (range, 28–76 years). The H&H and Fisher grades are shown in Table 1. Thirty-seven patients (97.4%) had a mild neurological condition (H&H grade 1–3), and 6 patients (15.8%) had a lesser hemorrhage (mFS 1 or 2).

**Table 1:** Patient and aneurysm characteristics.

	**All cases (*n* = 38)**
Female	22 (57.9)
Age (years)	51.0 (44.8–60.3)
Hypertension	18 (47.4)
Diabetes	4 (10.5)
Current smoking	17 (44.7)
**H&H grade**
I	12 (31.6)
II	24 (55.3)
III	4 (10.5)
IV	1 (2.6)
V	0 (0.0)
**Fisher grade**
1	5 (13.2)
2	1 (2.6)
3	18 (47.4)
4	14 (36.8)
Size of aneurysm	4.45 (2.97–13.85)
Neck diameter	3.83 (1.80–8.00)
**Aneurysm shape**
Saccular	14 (36.8)
Blister	12 (31.6)
Dissecting	12 (31.6)
**Aneurysm location**
ICA: ophthalmic	8 (21.1)
ICA: cavernous sinus	2 (5.3)
ICA: posterior communicating	17 (44.7)
Middle cerebral artery: M1	1 (2.6)
Anterior cerebral artery:A3	1 (2.6)
Vertebral artery	4 (10.5)
Basilar artery	4 (10.5)
Vertebrobasilar artery	1 (2.6)
Perforator involving aneurysm	10 (26.3)

*Variables are expressed as the median (interquartile range) or number of patients (%)*.

The aneurysms included 14 (36.8%) saccular IAs, 12 (31.6%) blister-like IAs, and 12 (31.6%) dissecting IAs. Twenty-nine (76.3%) IAs were located in the anterior circulation, and 9 (23.7%) were in the posterior circulation. The median size was 4.45 mm (range, 1.41–26.9 mm). Perforator involvement was noted in 10 (26.3%) IAs, and 2 of the 38 patients (5.3%) had additional aneurysms.

### Treatment

The Classical PED and PED Flex were used in an equal number of cases (50.0 vs. 50.0%). All cases except one (97.4%) involved placement of one PED. The median time from SAH onset to PED placement was 10.2 days (range, 2–45 days). Early PED placement ( ≤ 15 days) was performed in 27 (71.1%) cases. Adjunctive coiling was performed in 22 (57.9%) cases, while PED placement was performed as a stand-alone procedure in the remaining cases (42.1%). Complete neck coverage was successfully achieved in all cases. Two patients (5.3%) experienced inadequate expansion during PED deployment, requiring balloon angioplasty; one patient had inadequate expansion, which led to acute in-stent thrombosis and death. Immediate complete aneurysm obliteration was achieved in 13 cases (34.2%), and incomplete aneurysm obliteration was noted in 25 cases (65.8%) [including 2 neck remnants (5.3%) and 23 residual aneurysms (60.5%)]. Significant contrast stasis in the venous phase was noted in 22 cases (65.6%).

### Complications

Stroke-related complications (hemorrhagic and ischemic) during hospitalization were observed in 12 patients (31.6%). Among them, procedure-related hemorrhagic complications were noted in 4 patients (10.5%), including 2 with saccular aneurysms and 1 with a blister aneurysm with rebleeding during the procedure. All of these patients died within 1 month. Intraparenchymal hemorrhage was discovered in one case during the procedure, which was caused by perforating vessel injury. Conservative treatment was performed, and the patient exhibited a favorable outcome at the last follow-up. Symptomatic procedural-related infarction was noted in six cases (15.8%) during hospitalization. The cause in one case of a basilar artery dissecting aneurysm was inadequate PED expansion followed by acute in-stent thrombosis. The aneurysm was not obliterated after PED deployment; therefore, intraoperative thrombolysis was not administered. Although external ventricular drain (EVD) placement was performed, this patient died from brainstem and occipital lobe infarction 10 days later. The reason for infarction in one case of a blister-like aneurysm and another case of dissection of the basilar artery may be perforator infarction caused by PED implantation. Procedural-induced or aggravated cerebral vasospasm may have caused infarction in three other cases. Symptomatic non-procedure-related (SAH-related) infarctions occurred in two cases (5.3%) during hospitalization. One occurred before PED implantation, and the other occurred 3 days after PED implantation, which was not associated with PED implantation and may have been caused by delayed cerebral vasospasm.

Non-stroke-related complications were observed in four (10.5%) patients during hospitalization. One patient (2.6%) experienced a direct carotid cavernous fistula, which was completely obliterated through a transvenous approach. Two patients (5.3%) had symptomatic hydrocephalus and underwent transient EVD placement after embolization. Another patient (2.6%) also had symptomatic hydrocephalus but underwent transient EVD placement and then ventriculoperitoneal shunt placement after embolization. However, this patient died 1 month later because of intracranial infection. Although two other patients underwent EVD placement after intraoperative rupture, no EVD-related hemorrhage occurred in this study.

### Clinical and Angiographic Outcomes

The mortality rate was 13.2% (5/38) in our study; three patients died from intraoperative rupture, one patient died from intraprocedural in-stent thrombosis, and the other patient died from intracranial infection after ventriculoperitoneal shunt placement. New symptomatic cerebral infarction occurred in two cases (4.3%) during follow-up. One case involved a saccular aneurysm in the ophthalmic segment of the internal carotid artery 4 months after the procedure, possibly caused by mild in-stent stenosis, and the symptoms were completely relieved with conservative treatment. The other case involved a basilar artery dissecting aneurysm, and the patient exhibited symptoms of brainstem perforator infarction 15 days after PED placement. The patient also exhibited a favorable outcome at the last follow-up. Platelet function tests were performed on both patients, and the results indicated sufficient responses to clopidogrel and aspirin. Thirty-two patients (84.2%) had favorable outcomes (mRS score 0–2), and 6 patients (15.8%) had unfavorable outcomes at the last follow-up. The mRS scores were distributed as follows: 19 (50.0%) patients had an mRS score of 0, 10 (26.3%) had an mRS score of 1, 3 (7.9%) had an mRS score of 2, 1 (2.6%) had an mRS score of 3, and 5 (13.2%) had an mRS score of 6.

Angiographic follow-up data were available for 32 of 33 surviving patients (97.0%) at 3–30 months (median time, 6 months), including 29 who underwent digital subtraction angiography, 2 who underwent computed tomography angiography, and 1 who underwent magnetic resonance angiography. Twenty-seven aneurysms (84.4%) exhibited complete occlusion, and 5 (15.6%) exhibited incomplete obliteration, including 2 (6.3%2) neck remnants and 3 (9.4%) residual aneurysms. No rebleeding occurred after PED placement, and mild in-stent stenosis was only noted in one case with a saccular aneurysm in the ophthalmic segment of the internal carotid artery 4 months later, which did not warrant further intervention.

### Factors Related to Clinical and Angiographic Outcomes and Procedure-Related Stroke

In this study, demographic factors, medical comorbidities, H&H grade, mFS grade, rebleeding before treatment, aneurysm shape (sac or non-sac), location (anterior or posterior circulation), size, perforator involvement, adjunctive coiling, and non-stroke-related complications were not associated with the clinical outcome (*p* > 0.05). Additionally, early PED placement ( ≤ 15 days) was not associated with a favorable outcome (Table 2). Hemorrhagic complications, but not infarction, were significantly associated with an unfavorable outcome in the univariate analysis (*p* = 0.009). The univariate analysis also indicated a trend of an association of symptomatic hydrocephalus requiring shunting with an unfavorable outcome (*p* = 0.059). The multivariate analysis revealed that hemorrhagic complications were an important predictor of an unfavorable outcome (*p* = 0.003) [odds ratio, 90.0; 95% confidence interval (CI), 4.413–1,835.392]. All four cases of hemorrhagic complications were procedure-related complications, and three patients (75%) died of intraoperative aneurysm rupture during follow-up.

**Table 2:** Factors associated with the clinical outcome.

	**mRS**		***p***
	**0–2 (*n* = 32)**	**3–6 (*n* = 6)**	
Female	19 (50.0)	3 (7.9)	0.682
Age (≥51 years)	16 (42.1)	3 (7.9)	1.000
Smoking	14 (36.8)	3 (7.9)	1.000
Drinking	4 (10.5)	0 (0.0)	1.000
Diabetes	4 (10.5)	0 (0.0)	1.000
Hypertension	15 (39.5)	3 (7.9)	1.000
Rebleeding before treatment	2 (5.3)	1 (2.6)	0.412
H&H grade (4–6)	0 (0.0)	1 (2.6)	0.158
mFS grade (3–4)	26 (71.1)	6 (13.2)	0.562
Aneurysm of anterior circulation	25 (65.8)	4 (10.5)	0.613
Sac aneurysm	10 (26.3)	3 (7.9)	0.392
Aneurysm size (>7 mm)	11 (28.9)	4 (10.5)	0.188
Perforator involving aneurysm	7 (18.4)	3 (7.9)	0.310
Early treatment ( ≤ 15 d)	22 (57.9)	5 (13.2)	0.650
Classic PED	17 (44.7)	2 (5.3)	0.660
Additional coiling	18 (47.4)	4 (10.5)	1.000
Stroke complications	8 (21.1)	4 (10.5)	0.066
Hemorrhage complications^*^	1 (2.6)	3 (7.9)	0.009
Infarction complications	7 (18.4)	1 (2.1)	1.000
Non-stroke complications	2 (5.3)	2 (5.3)	0.110
Hydrocephalus required shunting	1 (2.6)	2 (5.3)	0.059
Carotid cavernous fistula	1 (2.6)	0 (0.0)	1.000
Procedural-related stroke complications^*^	6 (15.8)	4 (10.5)	0.031

* *Statistically significant*.

Factors related to overall procedure-related stroke (hemorrhagic and infarction) during hospitalization were also investigated in our study. As revealed in Table 3, aneurysm size, perforator involvement, and classical PED placement were not associated with procedure-related hemorrhage and infarction in this study. According to univariate analysis, early PED placement was significantly associated with procedure-related stroke (*p* = 0.037). All cases of procedure-related hemorrhage and infarction occurred in patients with early PED placement. Adjunctive coiling was also associated with procedure-related stroke in the univariate analysis (*p* = 0.025). The procedure-related stroke rate was higher in patients with adjunctive coiling than in patients without additional coiling [40.9% (9/22) vs. 6.3% (1/16)]. However, a trend toward an association of adjunctive coiling with procedure-related hemorrhage or infarction was noted only in the multivariate analysis (*p* = 0.073) (odds ratio, 8.0; 95% CI, 0.822–77.818). Adjunctive coiling was not associated with procedure-related hemorrhage or infarction in the univariate analysis (*p* > 0.05).

**Table 3:** Factors associated with overall procedure-related stroke during hospitalization.

	**Overall procedural stroke complications**		***p***
	**No (*n* = 28)**	**Yes (*n* = 10)**	
Female	16 (42.1)	6 (15.8)	1.000
Age (≥51 years)	16 (42.1)	3 (7.9)	0.269
Smoking	12 (31.6)	5 (13.2)	0.727
Diabetes	4 (10.5)	0 (0.0)	0.556
Hypertension	14 (36.8)	4 (10.5)	0.719
H&H grade (4–6)	0 (0.0)	1 (2.6)	0.263
mFS grade (3–4)	25 (65.8)	7 (18.4)	0.310
Aneurysm of anterior circulation	21 (55.3)	8 (21.1)	1.000
Sac aneurysm	10 (26.3)	3 (7.9)	1.000
Aneurysm size (>**7** mm)	11 (28.9)	4 (10.5)	1.000
Perforator involving aneurysm	9 (23.7)	1 (2.6)	0.236
Early treatment ( ≤ 15 days)^*^	17 (44.7)	10 (26.3)	0.037
Classic PED	12 (31.6)	7 (18.4)	0.269
Additional coiling^*^	13 (34.2)	9 (23.7)	0.025

* *Statistically significant*.

Factors related to complete occlusion were also examined in 32 patients with available follow-up angiography data. As revealed in Table 4, aneurysm size, additional coiling, and aneurysm contrast filling (<5%) were not associated with complete aneurysm occlusion during follow-up. Contrast stasis in the venous phase was significantly associated with complete occlusion in the multivariate analysis (*p* = 0.050) (odds ratio, 15.2; 95% CI, 1.004–232.346). A trend of an association of perforator involvement with incomplete occlusion was also noted in this study (*p* = 0.058) (odds ratio, 12.05; 95% CI, 0.915–158.695).

**Table 4:** Factors associated with angiographic occlusion in cases with final follow-up angiography data available.

	**Angiographic occlusion**		***p***
	**Incomplete (*n* = 5)**	**Complete (*n* = 27)**	
Female	4 (12.5)	14 (43.8)	0.355
Age (≥52 years)	4 (12.5)	12 (37.5)	0.333
Smoking	1 (3.1)	12 (37.5)	0.625
Diabetes	1 (3.1)	3 (9.4)	0.512
Hypertension	1 (3.1)	14 (43.8)	0.338
H&H grade (4–6)	0 (0)	0 (0)	—
mFS grade (3–4)	5 (15.6)	22 (68.8)	0.564
Aneurysm of anterior circulation	4 (12.5)	20 (62.5)	1.000
Sac aneurysm	2 (6.3)	7 (21.9)	0.604
Aneurysm size (>7 mm)	2 (6.3)	9 (28.1)	1.000
Perforator involving aneurysm	3 (9.4)	4 (12.5)	0.057
Early treatment ( ≤ 15 days)	4 (12.5)	17 (53.1)	0.637
Classic PED	1 (3.1)	15 (46.9)	0.333
Additional coiling	3 (9.4)	15 (46.9)	1.000
**Immediate occlusion degree**
Significant contrast stasis^*^	1 (3.1)	21 (62.5)	0.037
Aneurysm contrast filling (<5%)	1 (3.1)	14 (43.8)	0.338

* *Statistically significant*.

## Discussion

The overall ischemic and hemorrhagic complication rate (31.6%) and the mortality rate (13.2%) were high in this study. A previous study also found an overall complication rate of 30.6% for ruptured IAs, but a rate of 14.6% for unruptured IAs (6). A recent multicenter study indicated a much higher procedural complication rate (45%), with combined ischemic and hemorrhagic complication rates up to 36% for acutely ruptured IAs (11). In this study, the procedural hemorrhagic and ischemic complication rates were 10.5 and 15.8%, respectively. Procedure-related stroke, especially hemorrhagic stroke, was the only predictive factor of an unfavorable outcome in our study. Only one literature review examined PED treatment of ruptured IAs; it found a high symptomatic neurologic complication rate (16.5%) (thromboembolic complications, 6.5%; hemorrhagic complications, 10%) (12). Flow diverter deployment is often technically demanding and may be more challenging for ruptured IAs with spasmodic vessels. In addition to the high incidence of complications, deployment failure and incomplete deployment were also frequently noted (13). Approximately 5% of cases exhibited incomplete PED deployment and required balloon dilatation in this study. Postoperative stent migration, in-stent stenosis, and perforator infarct are also other issues worth considering (14), and some patients even presented ischemic events after a long follow-up period (>1 year) (15). Therefore, a flow diverter may not be the first choice for treatment of acute ruptured aneurysms. However, flow diverters could reduce the possibility of aneurysm rebleeding because of a satisfactory rate of aneurysm occlusion. Therefore, the use of flow diverters for treatment of ruptured aneurysms should be carefully evaluated by neurosurgeons and neuroradiologists. Flow diverters may be more beneficial for complex IAs such as blister, dissecting, and giant or wide-necked aneurysms, which are difficult to treat with conventional endovascular and surgical approaches. However, increasing clinical experience and advances in vascular access systems and PED deployment may further decrease complications and extend the indications of flow diverters. Recently, new flow diverters with surface modifications (such as PED Shield technology and P48 and P64 hydrophilic polymer-coated flow diverters) have been used to treat acute ruptured aneurysms (16, 17), which might decrease the risk of dual antiplatelet therapy-related hemorrhagic complications and aneurysm rebleeding.

Early treatment is important to avoid aneurysm rebleeding. However, a previous study found that the overall rate of hemorrhagic or ischemic complications was higher in cases treated within the acute phase (18). One meta-analysis also found that early PED deployment indicated a slightly greater risk of hemorrhagic complications and stroke/death (19). IAs were more likely to rupture during the acute period under dual antiplatelet therapy. Early PED placement also increases the potential risks of hemorrhagic complications during invasive procedures under dual antiplatelet therapy. Meanwhile, vasospasm during the acute period could also pose a significant challenge for device manipulation. PED placement could not only induce vasospastic vessel dissection but also aggravate vasospasm and induce thrombosis formation in the acute hypercoagulable phase after SAH. Delayed PED placement may likely decrease procedural stroke complications; however, this would increase the risk of a second SAH before treatment. Therefore, other authors have recommended a staged treatment strategy for complex ruptured IAs, which was shown to be safe and effective (3, 20). The aneurysm dome or rupture site is first coiled to avoid rupture, and then delayed PED placement is performed after recovery from the hemorrhagic event. In this study, early PED placement was not associated with a high rate of procedure-related stroke in the multivariate analysis; therefore, PED placement could be performed early to avoid aneurysm rebleeding. However, early PED deployment in some acute complex aneurysms may be difficult. The following factors should be considered for treatment of acute ruptured IAs with PED placement: the patient's neurological condition, vascular tortuosity, spasm severity, and aneurysm morphology. Early flow diversion is more suitable for blister, dissecting, or fusiform aneurysms or patients with mild neurological conditions (19), while initial coiling in the acute phase followed by staged flow diversion should be considered for complex and giant saccular ruptured aneurysms or patients with serious neurological conditions who require invasive procedures (19).

Previous studies found that additional coiling could result in a higher incidence of, and could predict, complete occlusion (21, 22). However, our study did not support this conclusion. One meta-analysis also found no difference in occlusion of acute ruptured IAs with or without additional coiling (23). Furthermore, aneurysms with or without additional coiling exhibited no difference in rebleeding risk (11). These differences in results may be caused by heterogeneity of aneurysms or the degree of coil packing (loose vs. dense packing). Adjunctive coiling may increase the difficulty of stent release and increase stroke-related complications (such as aneurysm rupture) and morbimortality (24). However, the use of adjunctive coiling may also be an indirect marker of aneurysm complexity. Adjunctive coiling could provide a scaffold for intrasaccular thrombosis. Furthermore, it could not only provide immediate dome protection to prevent aneurysm rebleeding but also interfere with blood flow and improve the complete occlusion rate during follow-up (22, 25). Therefore, we still advocate that, if possible, adjunctive coiling should be performed in acute ruptured aneurysms. Some authors suggest that loose rather than dense packing of the aneurysm dome is sufficient for ruptured IAs (18). However, loose packing might result in less contrast stasis and delayed aneurysm occlusion, which increases the risk of aneurysm rerupture. The rebleeding rate was ~2.1–11% after PED deployment in the literature, and most rebleeding occurred in larger aneurysms during the acute period following the initial bleed (11, 23). Postoperative dual antiplatelet therapy and other aggressive treatments, such as 3-H treatment, lumbar puncture, and continual cerebrospinal fluid drainage, could also increase the rebleeding risk. In our study, contrast stasis in the venous phase after PED deployment was significantly associated with complete obliteration during follow-up. Therefore, coil-assisted embolization with contrast stasis at least in the venous phase may help to avoid rebleeding and recurrence of ruptured aneurysms.

The complete occlusion rate for ruptured aneurysms in the literature was ~78–90% after flow diversion during follow-up (12, 18, 23, 26). Our study showed a similar complete occlusion rate (84.3%). Previous studies found that large and posterior circulation IAs and older age may be associated with incomplete occlusion of ruptured IAs (12, 18, 23). However, our study found that only immediate contrast stasis in the venous phase was associated with complete obliteration during follow-up. Furthermore, a trend of association of perforator involvement with incomplete occlusion was observed in this study. The predictive factors for incomplete occlusion, including unruptured aneurysms, have also been examined in several large studies, and the results were inconsistent. Adeeb et al. reported 465 aneurysms treated with the PED and found that age > 70 years, non-smoking status, and shorter follow-up time were associated with incomplete occlusion (27). Daou et al. found that age > 65 years, prior stent placement, aneurysms in the distal anterior circulation, and longer follow-up duration were associated with incomplete occlusion (28). Madaelil et al. found that adjunctive coiling and multiple device constructs were associated with higher complete occlusion rates (25). Matthew et al. found that larger aneurysms and perforator involvement predicted incomplete occlusion, while adjunctive coiling predicted complete occlusion (22). In our study, contrast stasis in the venous phase after PED deployment was significantly associated with complete obliteration during follow-up. In addition, adjunctive coiling could disturb intra-aneurysmal flow and cause contrast stagnation. Furthermore, multiple PED deployment would increase stent metal coverage, decrease intra-aneurysmal pressure, and reduce blood flow into the aneurysm, which might reduce the rerupture and residual rates (25, 29). However, results regarding multiple PED deployment are not consistent. Furthermore, the first released PED might affect or hinder the release of the second PED and increase procedure-related complications. Meanwhile, increased metal coverage might increase the risk of side vessel occlusion and ischemic complications (6, 23, 30). The PED is also expensive and is not covered by medical insurance in China; therefore, overlapping PED placement is not recommended and was not frequently performed in our study.

There is no standardized recommendation on the use of antiplatelet therapy before and after placing flow diverters for ruptured IAs. The antiplatelet agents, dosage, and duration vary across different institutions. A comprehensive consideration should include the hemorrhagic and thrombotic risks, presence of coexisting residual aneurysms, and individual bioavailability of antiplatelet drugs. Patients with SAH are usually in a hypercoagulable state and prone to thromboembolic complications during stent implantation. Conventional antiplatelet drugs, such as aspirin and clopidogrel, have slow onset and a long half-life. Furthermore, early application of dual antibodies may reduce the risk of thrombosis but also increase the risk of aneurysm rupture and cerebral hemorrhage. New antiplatelet drugs, such as cangrelor, which presents a fast and reliable onset of action and reversible platelet inhibition without evidence of resistance, may be a suitable alternative to oral antiplatelet drugs in the acute phase of SAH (31). The intracranial hemorrhagic complication rate was ~8% in patients who underwent stent-assisted coiling for acutely ruptured IAs (32); therefore, EVD placement before initiating antiplatelet medication is recommended to avoid hemorrhage (14). The overall incidence of ischemic complications was 5% (33), and the incidence of in-stent stenosis ranged from 3.5 to 10% for IAs with flow diversion (34, 35). Some patients still presented ischemic events after flow diverter implantation even after a long follow-up period (>1 year) (15). Although early withdrawal of platelet therapy might increase the aneurism occlusion rate, prolonged antiplatelet therapy may be helpful to avoid ischemic events. Meanwhile, although the utility of platelet function testing remains controversial in interventional treatment of cerebrovascular disease, routine platelet function testing may identify non-responders and allow alternate dual-antiplatelet therapy to be provided to decrease the aforementioned ischemic complications. With the advancement of technology, flow diverters with antithrombogenic properties have greater prospects for application to decrease ischemic events in the future.

### Limitations

Although this was a multicenter study, PED placement as the primary treatment for acute ruptured IAs is not frequently performed in China, and this resulted in a small sample size. Meanwhile, this retrospective study has inherent selection bias, and the inclusion of heterogeneous aneurysms and those treated with or without additional coiling may affect the results. The follow-up period was short, and not all included patients had complete angiographic follow-up data. Therefore, some statistical error could be present, resulting in false-positive results or overestimation of the magnitude of an association. A prospective multicenter study with a larger sample size is needed to eliminate statistical variation. However, similar studies examining the treatment of ruptured aneurysms with flow diversion are still rare. Although the conclusion of our study was mainly based on our own experience, our multicenter retrospective study could still provide valuable information regarding the safety and efficacy of flow diverting stent placement in the treatment of acutely ruptured IAs.

### Conclusion

The PED is a feasible and effective treatment to prevent rebleeding and achieve aneurysm occlusion, but it is still associated with a substantial risk of periprocedural hemorrhagic and ischemic complications in patients with acute ruptured IAs. Therefore, the PED should be used selectively for acutely ruptured IAs. Additionally, adjunctive coiling might increase procedure-related stroke; however, it may reduce rebleeding of acutely ruptured IAs. Patients with immediate contrast stasis in the venous phase were more likely to achieve total occlusion. A prospective study with a larger sample size should be performed to verify our results.

## Data Availability Statement

The raw data supporting the conclusions of this article will be made available by the authors, without undue reservation.

## Ethics Statement

Ethical review and approval was not required for the study on human participants in accordance with the local legislation and institutional requirements. Written informed consent for participation was not required for this study in accordance with the national legislation and the institutional requirements.

## Author Contributions

WZ and HK: article drafting and writing. PZ: data collection and statistics. XY, BL, AM, YZ, DS, SG, HZ, YaW, DW, WS, and YuW: collection of original data. YuW: review this article. All authors contributed to the article and approved the submitted version.

## Funding

This study was sponsored by the National Natural Science Foundation of China (Grant Numbers: 81701160 and 81671139) and the National Key Research and Development Plan of China (Grant Number: 2016YFC1300800).

## Conflict of Interest

The authors declare that the research was conducted in the absence of any commercial or financial relationships that could be construed as a potential conflict of interest.

## Publisher's Note

All claims expressed in this article are solely those of the authors and do not necessarily represent those of their affiliated organizations, or those of the publisher, the editors and the reviewers. Any product that may be evaluated in this article, or claim that may be made by its manufacturer, is not guaranteed or endorsed by the publisher.

## Abbreviations

PED: pipeline embolization device
IAs: intracerebral aneurysms
SAH: subarachnoid hemorrhage
EVD: external ventricular drainage
H&H: Hunt and Hess
mFS: modified Fisher scale
mRS: modified Rankin Scale.
